# Mat Gold and How to Use It

**Published:** 1904-09-15

**Authors:** J. L. Young


### MAT GOLD AND HOW TO USE IT.

BY J. L. YOUNG, D.D.S.

There are several kinds of mat gold on the market. These golds were first introduced as crystal gold more than forty years ago. At that period there were several varieties, varying in kind only in the form of the crystals, produced by varying the methods in use for precipitating the gold from its solution. One form which was much valued was called sponge gold, and was made by several manufacturers.

Another was in thin layers of large crystalline structure; this was Leslie's crystal gold. Then there was Watt and Williams, and A. J. Watt's crystal gold, all similar, but yet unlike in microscopic appearance. The old crystal gold held popular favor for a number of years, but it was found that it could not be depended on for strength or integrity in the mass of filling. At about this period came the innovation of cohesive foil, which produced results differing so much in integrity of the mass of the filling, that crystal golds were forgotten in the attempts to restore contours by the use of cohesive foils and the methods of operating developed by such men as Corydon Palmer, Wm. H. Atkinson and Marshall H. Webb.

The present era of mat gold fillings began with the introduction of what is now known as "Solila Gold," made first by Dr. de Trey, of Switzerland. It is a matter of interest to note here that Dr. de Trey got his idea of mat gold from Dr. Williams, who was formerly associated with Dr. George Watt, of Xenia, Ohio, and they made what was then known as Watt and William's crystal gold. Dr. Williams went to Europe to practice and it was there that he met Dr. de Trey and probably did much to encourage him in developing his mat gold.

The principal golds of this form now on the market are de Trey's, White's moss fibre, A. J. Watt's crystal and Vernon's mat gold.

The reasons why I use the Vernon's mat gold—which works best in my hands—may be stated as follows: First, because I believe I can make a better filling with this form of gold than any other that I have used. Second, I do not tire my patients or hurt them so much, as I can work this foil with less cavity preparation in the way of seat for the filling and undercuts for retention; and, thirdly, because I can do more work with it than any other form of gold. That is to say, I can pack it more rapidly and safely than any of the foil praparations.

As to where it should be used, I should say that any cavity, no matter where it is located that can be filled with other forms of gold, can be filled with mat gold. And many cavities into which it would be impracticable to pack foil, this gold can be introduced with ease. There are not many cavities of any extent that cannot be filled successfully with this form of gold. The extreme distal cavities are not easily filled with it, nor can they be with any other form of gold. But by means of the matrix and hand pressure, the general run of the cavities may all be successfully filled without great fatigue.

To introduce this gold and get the best results, the cavities should be so formed that a sufficient pressure may be applied to condense the gold firmly against all tooth walls and cavity margins. The subject of cavity preparation is too large a one to discuss at this time; I would simply suggest that all decay should be removed, the cavity thoroughly sterilized, and the margins extended and made true, and if possible in the proximal cavities, the cavity margins should be made as nearly as is practicable with right angles to the surface of the tooth at the cavity margins. If it is a proximal cavity I then insert a matrix, if indicated, and when properly placed and the tooth sterilized, it is then ready for beginning the filling operation. I then take as large a piece of gold as can be carried into the cavity; this is condensed and the filling built upon it in successive layers. Care should be exercised to properly anneal the gold, not in the flame of the lamp, as this would discolor or burn it, but the various forms of electric annealers or porcelain disks over Bunsen flames for annealing should be used.

This gold should always be annealed to a low red color to make it thoroughly cohesive. I sometimes use a rope of soft gold foil, or gold and tin, to start the filling. This should be large enough to fill the cervical part of the cavity, and should then be carefully forced to place and condensed. I prefer to condense with hand pressure. As to the use of tin and gold I find that by combining tin with the gold it does not turn so black. I use the foil to start the filling with, because it works easily and makes a good adaptation to the margins and cavity seat.

I find that tin and gold rolled together produce a gray color instead of a black one if tin alone be used, and the combination seems harder than when they are used singly. The tin is used for its therapeutic effect on the dentine and because it works easier than foil. After laying into the cavity a sufficient foundation of the foils, I then begin and pack the rest of the cavity with the Vernon's mat gold, annealing it well and condensing with finely serrated pluggers of suitable forms to carry it into every part of the cavity. While this gold is quite cohesive when well annealed, it also flows under pressure so that it can be spread laterally against the walls or into considerable undercuts.

In proximal cavities where a matrix must be used, I first prepare the cavity extending laterally as much as is necessary and then place the matrix and fasten to place. In these cavities an acute angle is formed on the lateral margins by the matrix and lateral walls into which it is troublesome to get the mat gold condensed thoroughly. To accomplish this object successfully, after laying in the bottom of the filling with gold, or gold and tin, I take a cylinder of soft gold of the proper length and set it up on end in this acute angle and set it into place somewhat loosely. I then begin packing the cavity with mat gold, which, as it is carried against this cylinder of foil, drives it tightly into the farthest part of the angle, and the mat gold uniting cohesively with it, condenses, making a perfect adaptation to the cavity margin and one that will not leak and easily break away. By using a kind of rotary force, the mat gold is directed into the angle and carries before it the foil.

As a principle, I think it is highly important that we restore occlusal contours as well as lateral contours. A flat, occlusal surface, and especially, should it slant toward the proximal surface, will not only be a bad thing because it directs the food into the inter-dental space, but it may interfere with the proper occlusion of the teeth and be the means of causing an unfortunate mal-occlusion. I also believe in making hard and full contours on the proximal surfaces, to prevent the teeth from coming together and thus disturbing proper occlusal relations. This is really one of the great faults of amalgam fillings, when placed in the proximal surfaces. The amalgam filling does not set hard quick enough to maintain a rigid surface, so that when the separator or matrix is removed, the teeth come together and the fillings flatten, and there is no extra material to dress off when the fillings have completely hardened and still leave the proximal contact points as full as they should be

In small cavities in the incisor teeth there is no need to make extensive undercuts to retain the filling when using mat gold. A small anchorage toward the cervical and another toward the incisal ends of the cavity will be all that is required. Then place a proper sized piece of gold in each of these undercuts and condense; then lay in a piece which will extend from one anchorage to the other and will cover the lingual walls of the cavity, and condense it to place. This will not only form the lingual part of the filling, but will also fix the anchorages at the cervical and incisal ends of the cavity. The rest of the filling can then be easily completed.

